# Chronic HCV infection promotes cytotoxicity in antigen-specific CD8^+^ T cells regardless of virus specificity

**DOI:** 10.3389/fviro.2023.1198361

**Published:** 2023-06-27

**Authors:** Ana C. Maretti-Mira, Matthew P. Salomon, Angela M. Hsu, Chikako Matsuba, Lucy Golden-Mason

**Affiliations:** 1 USC Research Center for Liver Diseases, Keck School of Medicine, University of Southern California, Los Angeles, CA, United States; 2 Division of Gastrointestinal and Liver Diseases, Department of Medicine, Keck School of Medicine, University of Southern California, Los Angeles, CA, United States

**Keywords:** chronic HCV, resolved HCV, CMV, flu, dextramer, scRNAseq

## Abstract

**Introduction:**

Despite advancements in hepatitis C virus (HCV) infection treatment, HCV still represents a significant public health burden. Besides progressive hepatic damage, viral persistence has lasting effects on innate and adaptive immune responses. Lack of a complete understanding of the factors driving an effective HCV response contributes to the failure to develop a vaccine for prevention. This study advances the existing knowledge on HCV-specific CD8^+^ T cells and describes the impact of current or past HCV infection on CD8^+^ T cells specific for other viruses.

**Methods:**

We used barcoded-dextramers to identify and sort CD8^+^ T cells specific for HCV, cytomegalovirus, and influenza, and characterized them using single-cell RNA sequencing technology. Our cohort included chronic (cHCV), spontaneously resolved (rHCV), and subjects undergoing direct-acting antiviral (DAA) therapy.

**Results:**

We show that HCV-specific CD8^+^ T cells have cytotoxic features in patients with cHCV, which is progressively reduced with DAA therapy and persists 12 weeks after treatment completion. We also observe a shift in the CD8^+^ T cell phenotype on DAA treatment, with decreased effector memory and exhausted cell signatures. In rHCV, we also detected a smaller proportion of effector memory cells compared to cHCV. The proportion of CD8^+^ exhausted T cells in cHCV and rHCV subjects was comparable. Moreover, we also observed that non-HCV virus-specific CD8^+^ T cells exhibit robust cytotoxic traits during cHCV infection.

**Discussion:**

Altogether, our findings suggest that cHCV infection promotes cytotoxicity in CD8^+^ T cells regardless of virus specificity. The immunological changes caused by cHCV infection in CD8^+^ T cells may contribute to worsening the ongoing hepatic damage caused by HCV infection or exacerbate the immune response to possible co-infections. Our data provide a resource to groups exploring the underlying mechanisms of HCV-specific T cell spontaneous and treatment-induced resolution to inform the development of effective vaccines against HCV infection.

## 1 Introduction

Hepatitis C virus (HCV) infection is the most common bloodborne infection reported in the United States. Between 15% and 42% of acute HCV infection cases can resolve spontaneously (rHCV) (1). However, the viral persistence observed in the majority of infected subjects leads to a chronic infection (cHCV), which can now be successfully treated using direct-acting antiviral (DAA) therapy (2). The current DAA regimens are highly effective, well tolerated, typically require only 8–12 weeks of treatment, and have been broadly used over the past decade (3). Nevertheless, HCV infection still represents a significant cause of liver-related morbidity, transplantation, and mortality, offering substantial challenges for disease prevention, control, and elimination (1, 4). Despite advancements in HCV infection treatments, a preventative vaccine has not yet been developed. Barriers to HCV vaccine development are significant and include an incomplete understanding of protective immune responses. Although the individuals that spontaneously clear HCV infection are susceptible to reinfection, they display a faster virus clearance during the secondary HCV infection with rare progression to chronicity, suggesting the development of an efficient adaptive immune response during the spontaneous resolution of the primary HCV infection that depends on HCV-specific T cells (5). Therefore, comprehending the underlying mechanisms of HCV-specific T cell spontaneous and treatment-induced resolution is critical to inform the development of effective vaccines against HCV infection.

The HCV-specific CD4^+^ and CD8^+^ T cell crosstalk drives the immune response against HCV infection. While virus-specific CD4^+^ T cells participate in the spontaneous resolution of the infection by facilitating the priming of CD8^+^ T cells, HCV-specific CD8^+^ T cells can rapidly terminate virus replication (6, 7). Cytotoxic CD4^+^ T cells can also be found at a much lower frequency during active viral hepatitis, mainly in patients with elevated levels of aspartate aminotransferase (AST), indicating that cytotoxic CD8^+^ T cells are the main cytolytic effectors for viral control (8). However, the phenotype and functionality of HCV-specific CD8^+^ T cells are remarkably impaired during cHCV infection, ultimately failing to clear the virus and contributing to the ongoing liver disease (9). In addition to the progressive liver damage caused by cHCV, accumulating evidence supports that cHCV infection can also impair global innate and adaptive immune responses (10–13). However, the impact of previous or current HCV infection on virus-specific T cells against other viral infections such as cytomegalovirus (CMV) and influenza (Flu) is not fully understood.

In the present study, we aimed to identify the transcriptomic differences among HCV-specific CD8^+^ T cells during and after HCV infection and to determine the effect of current or past HCV infection on CD8^+^ T cells specific for other viruses. Here, we combined the use of barcoded-dextramers with single-cell RNA sequencing (scRNA-seq) to characterize circulating HCV-specific CD8^+^ T cells from cHCV and rHCV subjects and DAA-treated HCV patients and profile the CD8^+^ T cells specific for CMV and Flu from some of the same subjects.

## 2 Materials and methods

### 2.1 Patient selection

This study was approved by the University of Southern California Institutional Review Board (HS-18–00254). Written and oral consent was obtained from all enrolled subjects.

Blood samples were collected from subjects with untreated chronic HCV infection (cHCV, n=4), from subjects that spontaneously resolved HCV infection (rHCV, n=4), and from non-HCV-exposed and control subjects (CTR, n=3). We also included chronic HCV patients successfully treated for 12 weeks with direct-acting-antivirals (DAA) (n=3) that had blood collected before therapy (Pre-tx), at an early stage (2–4 weeks coinciding with non-detectable viral load) of DAA therapy (Early-tx), in a later phase (8 weeks) of therapy (Late-tx), and at 12 weeks after treatment conclusion (Post-tx). All patients experienced a sustained virologic response (Supplementary Table 1). All HCV subjects were infected with HCV genotype 1. Peripheral blood mononuclear cells (PBMCs) were isolated from the blood samples, viably frozen at 20–50 million cells/mL, and stored in liquid nitrogen for subsequent use. All subjects were previously screened for immunogenic response to CMV and Flu epitopes, and the HCV cohort was also evaluated regarding the HCV epitopes. Only the positive subjects were included in this study.

### 2.2 Sample preparation

**FIGURE 1:**
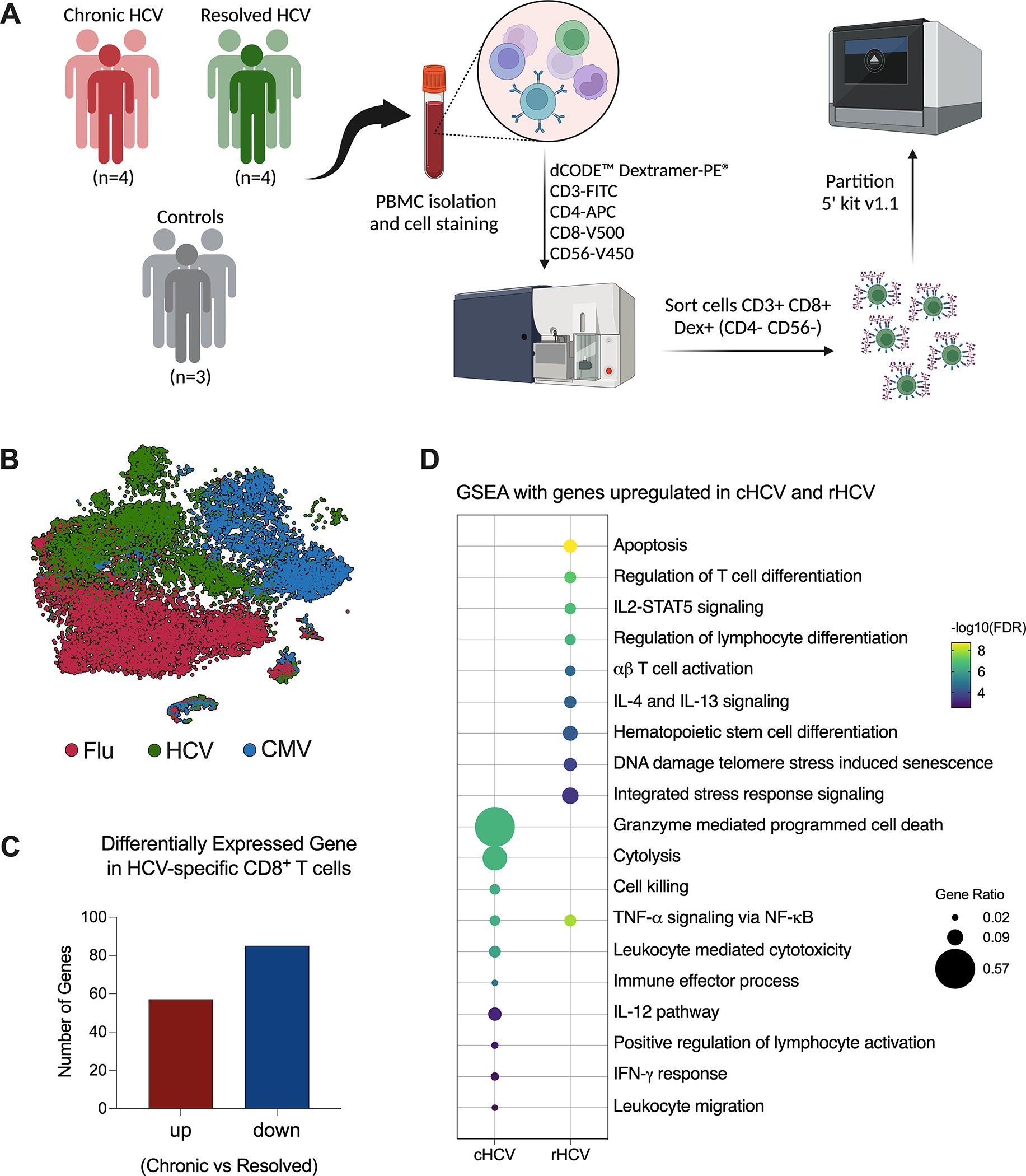
Investigation of virus-specific CD8^+^ T cells in chronic and spontaneously resolved HCV infection. **(A)** Experimental approach used for this study. Peripheral blood was collected from four patients with untreated chronic HCV infection (cHCV), four resolved HCV subjects (rHCV), and three non-HCV-exposed control subjects (CTR). Cells were stained with dCODE-Dextramers-PE (HCV A1_1435_, HCV A2_2594_, CMV, and Flu), plus an antibody cocktail for CD8^+^ T cell identification, then sorted and partitioned for single-cell RNAseq analysis. (Created with BioRender.com). **(B)** Integrated tSNE showing that CD8^+^ T cells were clustered according to dextramer binding into HCV (A1+A2 epitopes), CMV, and Flu. **(C)** Identification of differentially expressed genes (DEGs) in HCV-specific CD8^+^ T cells comparing cHCV and rHCV. **(D)** Most relevant Gene Set Enrichment Analysis (GSEA) terms enriched by genes upregulated in HCV-specific CD8+ T cells isolated from cHCV and rHCV.

PBMCs were thawed in RPMI media and centrifuged at 350×g for 10min at 4°C. Cells were washed with Washing Buffer (PBS, pH 7.4 + 5% FBS), centrifuged, and the cell pellet was resuspended in Stain Buffer (PBS, pH 7.4 + 5% FBS + 0.1g/L Herring sperm DNA). Then, we added 0.2 μl 100μM d-Biotin (Avidity LLC, Aurora, CO) per included dextramer and added 2μl of desired dCODE^™^ Dextramer^®^-PE reagents (Immudex, Denmark), including the negative control for allele and random dextramers (Table 1, dextramers #1–4 for cHCV and rHCV samples, and dextramer #1, #5–6 for DAA therapy samples). After 10 min of incubation in the dark, we added the antibody cocktail for CD8^+^ T cell staining (CD4-APC, CD3-FITC, CD8-V500, and CD56-V450) and incubated for 30 min at 4°C in the dark. We sorted the CD3^+^ CD8^+^ Dextramer^+^ cells using BD FACSAria^™^ Fusion into 90% FBS solution at 4°C (Figure 1A, Supplementary Figure 1). Sorted cells were centrifuged and resuspended in 50μl of PBS with 0.04% BSA for scRNAseq partitioning.

### 2.3 Single-cell RNAseq

Single-cell suspensions were processed using Chromium Next GEM Single Cell 5’ Kit v1.1 (10x Genomics, Pleasanton, CA) for samples from chronic and resolved HCV patients, and Chromium Next GEM Single Cell 5’ Kit v2 - dual index, for samples from cHCV patients treated with DAA therapy. Briefly, up to 16,500 cells were resuspended in the reaction mix and loaded into the Chromium Next GEM Chip G, followed by the loading of the Gel Beads and the partitioning oil. Chip G was loaded into the 10x Chromium controller for cell partitioning. After partitioning, 100μL of GEMs (Gel Bead-In EMulsion) were transferred to a new tube and placed at the C1000 Touch Thermal Cycler for the first phase of reverse transcription. All gene expression libraries and surface protein libraries were prepared and sequenced together to avoid batch effects. Single-cell RNAseq libraries were sequenced at the USC Norris Molecular Genomics Core and Keck Genomics Platform. Libraries were quantified using Kapa Quant Library qPCR (Roche Diagnostics Corporation, Indianapolis, IN), and quality was assessed using Agilent BioAnalyzer 2100. Prepared libraries were sequenced on Illumina Novaseq 6000 at 2×100 cycles. At least 40,000 reads were obtained per cell.

### 2.4 Data processing and analyses

Raw data processing: Cell by gene count matrices were generated by processing FASTQ files using the Cell Ranger count (version 5.0.1, 10X Genomics) pipeline with the GRCh38 (version refdata-gex-GRCh38-2020-A, 10X Genomics) human genome reference for each sample. For CD8^+^ T cell phenotype classification, we used signatures stablished by Hao et al. (14) and Doering et al. (15). Table 2 shows the genes included for each phenotype signature.

#### 2.4.1 Gene expression data pre-processing

##### Chronic vs. Resolved patients:

Gene expression data were processed using the R package Seurat (version 4) (16). We first, filtered the data for low quality cells by removing cells with 1) less than 500 reads, 2) less than 300 or greater than 2,000 genes detected, and 3) cells with greater than 10% of reads mapping to mitochondrial genes. We further filtered out cells with CD3 and CD8 expression less than 1. Dextramer positivity was determined by the maximum expressing dextramer within each cell. To account for any background non-specific dextramer staining we removed any cell with a maximum dextramer read count less than 10 times higher than the sum of all other dextramer counts. After filtering, individual samples were merged by disease state (i.e., Chronic, Resolved, and Control) and the three groups where integrated into a single unified data set using the SCTransform integration workflow implemented in Seurat (17, 18). Differentially expressed genes between each pair of disease states were detected using the FindMarkers function in Seurat using default parameters. A significance threshold of adjusted p-value < 0.1 was used for all tests. tSNE plots were visualized using functions from the SCPubr package (19). Gene signature values were calculated using the AddModuleScore function in Seurat.

##### DAA treated cHCV patients:

The samples obtained from DAA treated subjects were first filtered for low quality cells by removing cells with 1) less than 500 reads, 2) less than 300 or greater than 2,500 genes detected, 3) cells with greater than 20% of reads mapping to mitochondrial genes, and 4) cells with CD3 and CD8 expression less than 1. Dextramer positivity was determined by the maximum dextramer count within each cell and cells with the maximum dextramer count less than 10 times higher than the sum of all other dextramer counts were filtered out. Filtered cells were normalized and processed using functions in Seurat. Differentially expressed genes between each treatment time point were detected using the FindMarkers function in Seurat with default parameters and using an adjusted p-value > 0.1 as a significance threshold.

#### 2.4.2 Biological meaning

Ingenuity Pathway Analysis (IPA) software (v01-20-04, Qiagen) was used to determine the canonical pathways and biological processes altered in the different cell groups (20). The Gene Set Enrichment Analysis (GSEA) platform was used to identify the biological processes enriched by the differentially expressed genes (21).

## 3 Results

### 3.1 Identification of circulating virus-specific CD8^+^ T cells in chronic and spontaneously resolved HCV subjects

Acute HCV infection can either progress to chronicity (cHCV) or spontaneously resolve (rHCV). Accumulating evidence suggests changes in the adaptive immune response against other viruses during and after HCV infection. To identify differences that influence the fate of HCV infection, we isolated CD8^+^ T cells from the blood of four subjects with untreated chronic HCV and four subjects that resolved HCV without treatment. Here, we combined barcoded dCODE^™^ Dextramers and single-cell RNAseq (scRNAseq) technology (10x Genomics) to isolate and profile CD8^+^ T cells specific for the HCV epitopes ATDALMTGY_NS3:1435–1443_ (A1_1435_) or ALYDVVTKL_NS5:2594–2602_ (A2_2594_). To evaluate the effects of current and past HCV infection on the immune response against other viruses, we isolated from the same subjects CD8^+^ T cells specific for CMV (A2 - NLVPMVATV_pp65:495–504_) and Flu (A2 - GILGFVFTL_MP:58–66_). Three non-HCV-exposed subjects were included as controls. The dataset we generated allowed us to determine biologically relevant differentially expressed genes (DEG) and pathways influenced by HCV infection in HCV-, CMV- and Flu-specific CD8^+^ T cells in chronic and resolved HCV infection (Figure 1A).

After quality control and filtering out doublets and other cells not annotated as CD8^+^ T cells or not positive for Dextramer surface staining, we profiled a total of 5,121 A1_1435_-specific; 1,268 A2_2594_-specific; 10,177 CMV-specific; and 10,137 Flu-specific CD8^+^ T cells. The t-Distributed Stochastic Neighbor Embedding (tSNE) plot shows that the CD8^+^ T cells clustered according to virus specificity (Figure 1B). No significant differences were observed in the frequencies of the HCV clusters among cHCV and rHCV subjects (data not shown).

### 3.2 HCV-specific CD8^+^ T cells are more cytotoxic in chronic HCV subjects than in resolved HCV subjects

We evaluated the differences between HCV-specific CD8^+^ T cells in cHCV versus rHCV. We detected a total of 142 DEGs, where 57 genes were upregulated in cHCV and 87 were upregulated in rHCV (Figure 1C, Supplementary Table 2). The genes upregulated in cHCV enriched biological processes and pathways mainly related to cytotoxicity, such as cell death induced by granzymes, cytolysis, and cell killing, and other processes related to lymphocyte activation, such as IL-12 signaling and IFN- γ response. The genes upregulated in rHCV were related to cell viability (apoptosis, cell senescence, stress response) and cell differentiation and activation (Figure 1D). Our findings suggest that after spontaneous resolution, the cytotoxicity of HCV-specific CD8^+^ T cells is significantly reduced.

### 3.3 CD8^+^ T cells specific for different HCV epitopes demonstrate distinct profiles

**FIGURE 2:**
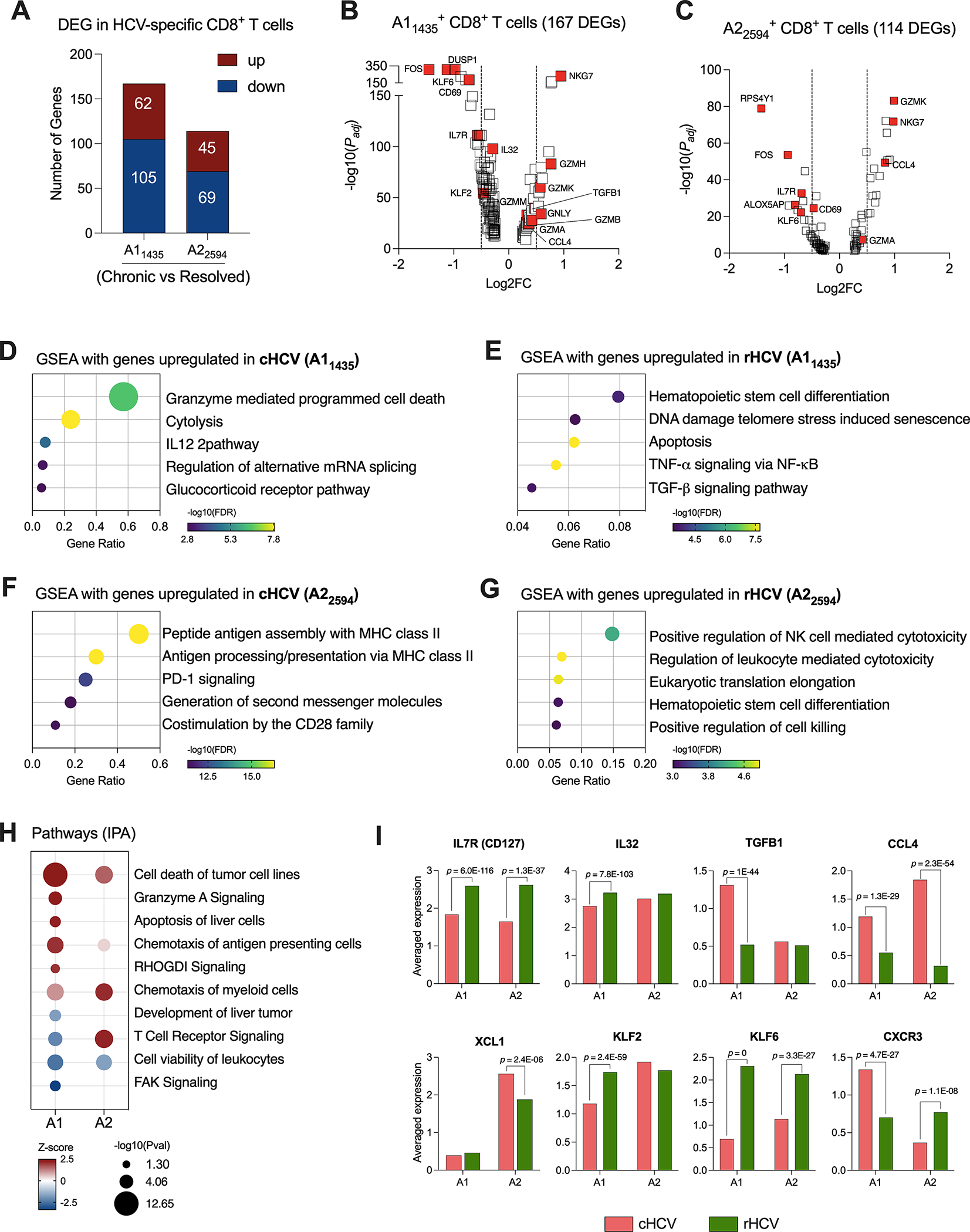
HCV-specific CD8^+^ T cells that recognize different HCV epitopes have different immunological profiles. **(A)** DEGs identified in HCV A1_1435_- and A2_2594_-specific CD8^+^ T cells comparing cHCV versus rHCV. **(B, C)** Volcano plots showing differentially expressed gene distribution in HCV-specific cells in cHCV patients compared to rHCV subjects. **(D, E)** Plots showing the most relevant pathways related to DEG upregulated in cHCV and rHCV in A1_1435_-specific cells. GSEA: Gene Set Enrichment Analysis. Bubble plot size derived from gene ratio located at X-axis. **(F, G)** Plots showing the most relevant pathways related to DEG upregulated in cHCV and rHCV in A2_2594_-specific cells. **(H)** Identification of pathways by Ingenuity Pathway Analysis (IPA) using DEG lists from A1_1435_- and A2_2594_-specific cells. **(I)** Genes with immunological relevance altered in A1_1435_- and A2_2594_-specific CD8+ T cells from cHCV and rHCV subjects.

For this study, we selected subjects that expressed the human leukocyte antigen (HLA) alleles HLA-A*0101 and HLA-A*0201, and we isolated CD8^+^ T cells specific for two HCV epitopes: A1_1435_ and A2_2594_. We evaluated the transcriptome of these cells by comparing cHCV to rHCV groups. In cHCV, we found 167 DEG in A1_1435_-specific CD8^+^ T cells (62 up and 105 downregulated), and 114 DEG in A2_2594_-specific CD8^+^ T cells (45 up and 69 downregulated) (Figure 2A, Supplementary Table 2). The DEGs of immunological relevance in A1_1435_- and A2_2594_-specific CD8^+^ T cells are shown in Figures 2B, C.

The genes of the A1_1435_-specific CD8^+^ T cells upregulated in cHCV were related to cytotoxicity (Figure 2D), while the genes upregulated rHCV were associated with cell differentiation and viability, such as apoptosis and senescence (Figure 2E). In contrast, the genes of the A2_2594_-specific CD8^+^ T cells upregulated in cHCV subjects were involved in antigen processing and presentation, and PD-1 and CD28 signaling (Figure 2F), whereas the genes upregulated in rHCV were related to cytotoxicity (Figure 2G).

We used the Ingenuity Pathway Analysis (IPA, Qiagen) platform to identify the most relevant pathways based on the balance between up and downregulated genes during chronic HCV infection (Figure 2H). We found that A1_1435_-specific CD8^+^ T cells in cHCV display a robust upregulation of cytotoxicity, apoptosis of liver cells, and chemotaxis of antigen presenting cells, with decreased TCR signaling and focal adhesion kinase (FAK) signaling. The A2_2594_-specific CD8^+^ T cells demonstrated strong TCR signaling and chemotaxis of myeloid cells. Both cell types showed decreased cell viability.

Among the most relevant genes encoding proteins with immunological function (Figure 2I, Supplementary Table 3), IL7R and KLF6 are upregulated in rHCV in A1 and A2 cells, while IL32 and KLF2 are upregulated in rHCV in the A1 group. We also found CCL4 upregulated in both A1 and A2 cells in cHCV, while TGFB1 is upregulated in the A1 group, and XCL1 is upregulated in the A2 group. Interestingly, CXCR3 is upregulated in the A1 group in cHCV and upregulated in the A2 group in rHCV.

Due to the significant functional changes observed in HCV-specific CD8^+^ T cells from cHCV compared to rHCV subjects suggested by our dataset, we investigated the overall HCV-specific CD8^+^ T cell phenotypes. For that purpose, we used the annotation established by Hao et al. (14) to determine naïve, central memory, and effector memory CD8^+^ T cells, and the dataset published by Doering et al. (15) was used to annotate CD8^+^ exhausted T cells (Table 2). The A1 and A2 HCV-specific CD8^+^ T cells displayed similar phenotype changes when comparing cHCV and rHCV (Supplementary Figures 2A, B). Resolved HCV subjects showed a higher proportion of HCV-specific naïve and central memory T cells, while effector memory T cells were predominant in cHCV. There was no significant difference in T exhausted HCV-specific T cells between cHCV and rHCV subjects.

### 3.4 DAA therapy reduces the cytotoxicity of HCV-specific CD8^+^ T cells

**FIGURE 3:**
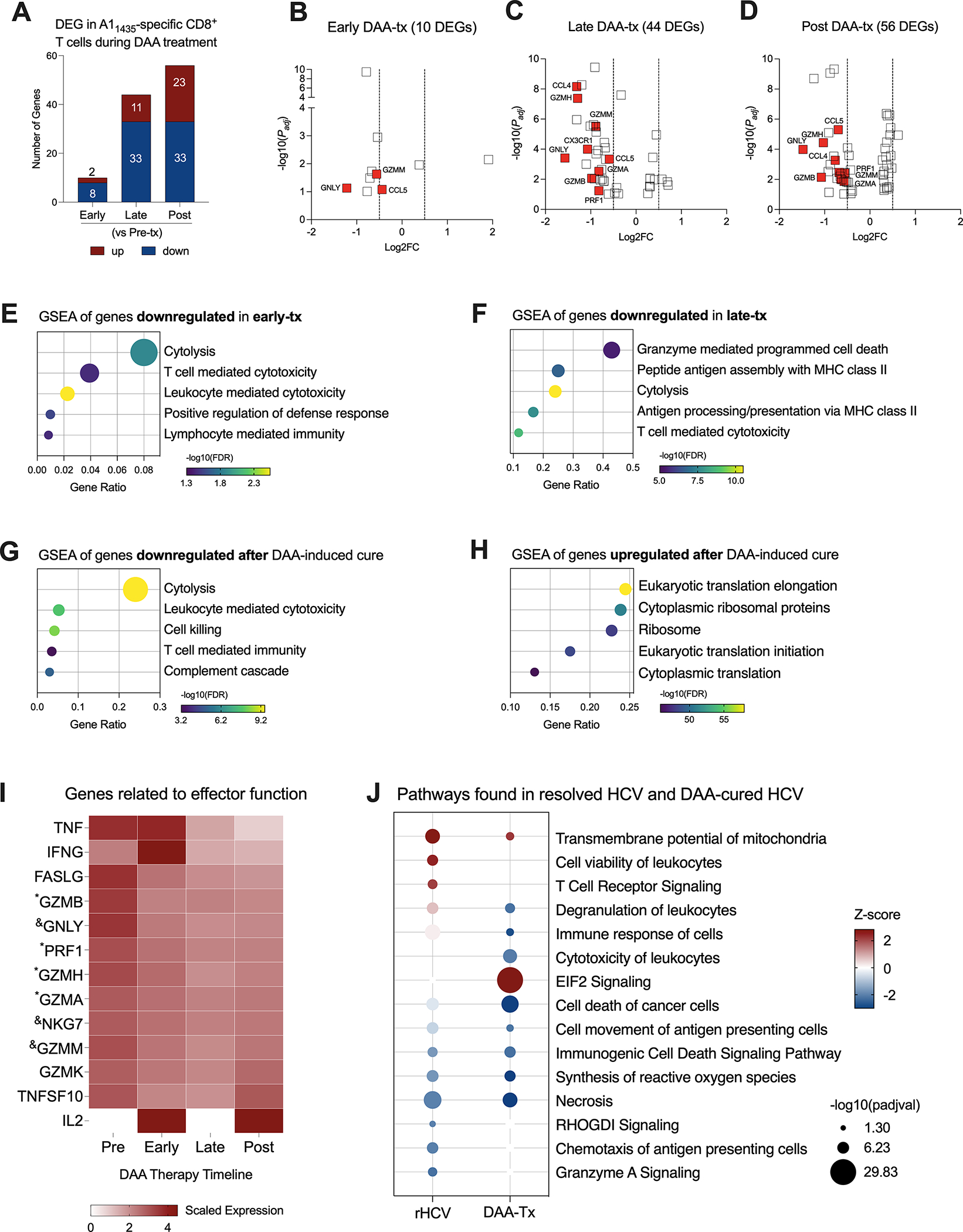
Evaluation of transcriptional changes in A1_1435_-specific CD8^+^ T cells during DAA therapy. **(A)** Differentially expressed genes identified in three stages of DAA treatment: early, late and post. **(B–D)** Volcano plots showing the distribution of DEG identified in early-, late-, and post- in comparison to pre-treatment (tx). **(E–G)** Plots show Gene Set Enrichment Analysis (GSEA) terms resulting from the enrichment analysis of genes downregulated in early- **(E)**, late- **(F)**, and post-tx **(G)**. Bubble plot size derived from gene ratio located at X-axis. **(H)** Plot showing GSEA terms resulting from the enrichment analysis of genes upregulated post-treatment. Bubble plot size derived from gene ratio located at X-axis. **(I)** Heatmap displaying the scaled expression of genes involved in CD8^+^ T cell effector functions in the different phases of DAA therapy. ^&^DEG found in early, late, and post-tx. *DEG found in late and post-tx. **(J)** Comparison of pathways and biological processes identified in A1_1435_-specific CD8^+^ T cells isolated from spontaneously resolved HCV (rHCV) infection and DAA-mediated resolution of HCV infection.

Direct-acting antiviral (DAA) therapy is highly effective, with standard cure rates above 95% (22). We evaluated three subjects, positive for the HLA-A*0101 allele, that were successfully treated with DAA therapy for 12 weeks. We collected blood samples from these subjects before therapy started (Pre-tx), at 2–4 weeks of therapy corresponding with viral clearance (Early-tx), towards the end of the therapy (Late-tx), and 12 weeks after DAA therapy conclusion (Post-tx). We isolated A1_1435_-specific CD8^+^ T cells and evaluated them by scRNAseq. We profiled 105 cells in Pre-tx, 533 cells in Early-tx, 231 cells in Late-tx, and 394 cells in Post-tx. We identified the DEGs resulting from DAA therapy in comparison to the Pre-tx samples (Figure 3A, Supplementary Table 2). In the initial stages, DAA therapy modified the expression of only 10 genes (2 up, and 8 downregulated). The most relevant downregulated genes were GZMM, GNLY, and CCL5 (Figure 3B and Supplementary Tables 2, 3). In later stages, we detected 44 DEGs (11 up and 33 downregulated genes), where the most relevant downregulated genes were CCL4, GZMH, GZMM, CX3CR1, CCL5, GNLY, GZMA, GZMB and PRF1 (Figure 3C, Supplementary Tables 2 and 3). Most of these genes remained downregulated after completion of DAA therapy (Figure 3D, Supplementary Table 2), which showed 56 DEGs (23 up and 33 downregulated).

Downregulation of cytotoxicity starts in the early stages of DAA therapy and is still detected 12 weeks after DAA therapy was completed (Figures 3E–G). In the late phases of DAA therapy, there is also downregulation of antigen processing and presentation, and after cure, we also detected inhibition of complement cascade. Twelve weeks after DAA-treatment, we detected upregulation of events related to RNA translation (Figure 3H). We also observed the expression of genes related to the effector functions of CD8^+^ T cells (Figure 3I). Although some of them were not part of the DEG lists, they showed decreased expression from the Pre-tx to Post-tx groups. Of note, IFNG showed a peak in the expression in the early phases of the therapy, while IL2 was not detected in the chronic subjects and started to be expressed at the beginning of the therapy, which suggests an activation of these cells in the early phases of therapy. Moreover, we observed a long-lasting gene expression inhibition induced by DAA therapy on 7 genes early during treatment (CCL5, MYL6, GZMM, NKG7, KLRD1, IGLV2–14, and GNLY) and on 11 genes late in treatment (HLA-DRB5, ANXA4, FCRL6, PRF1, GZMA, PLEK, ZEB2, GZMB, FGFBP2, GZMH, and CCL4) (Supplementary Table 3). These 18 genes remained downregulated at 12 weeks after the conclusion of DAA therapy.

We compared the most relevant biological events detected in rHCV and DAA-mediated resolution of HCV. TCR signaling is only activated in A1 HCV-specific CD8^+^ T cells from rHCV, which also displays upregulation of cell viability. A1 HCV-specific CD8^+^ T cells from DAA-mediated resolution of HCV demonstrated upregulation of EIF2 signaling, which is related to cytoplasmic translation and elongation, and downregulation of degranulation, immune response, and synthesis of reactive oxygen species (Figure 3J). Altogether, our findings suggest that the DAA therapy dampens HCV-specific CD8^+^ T cell effector functions.

**FIGURE 4:**
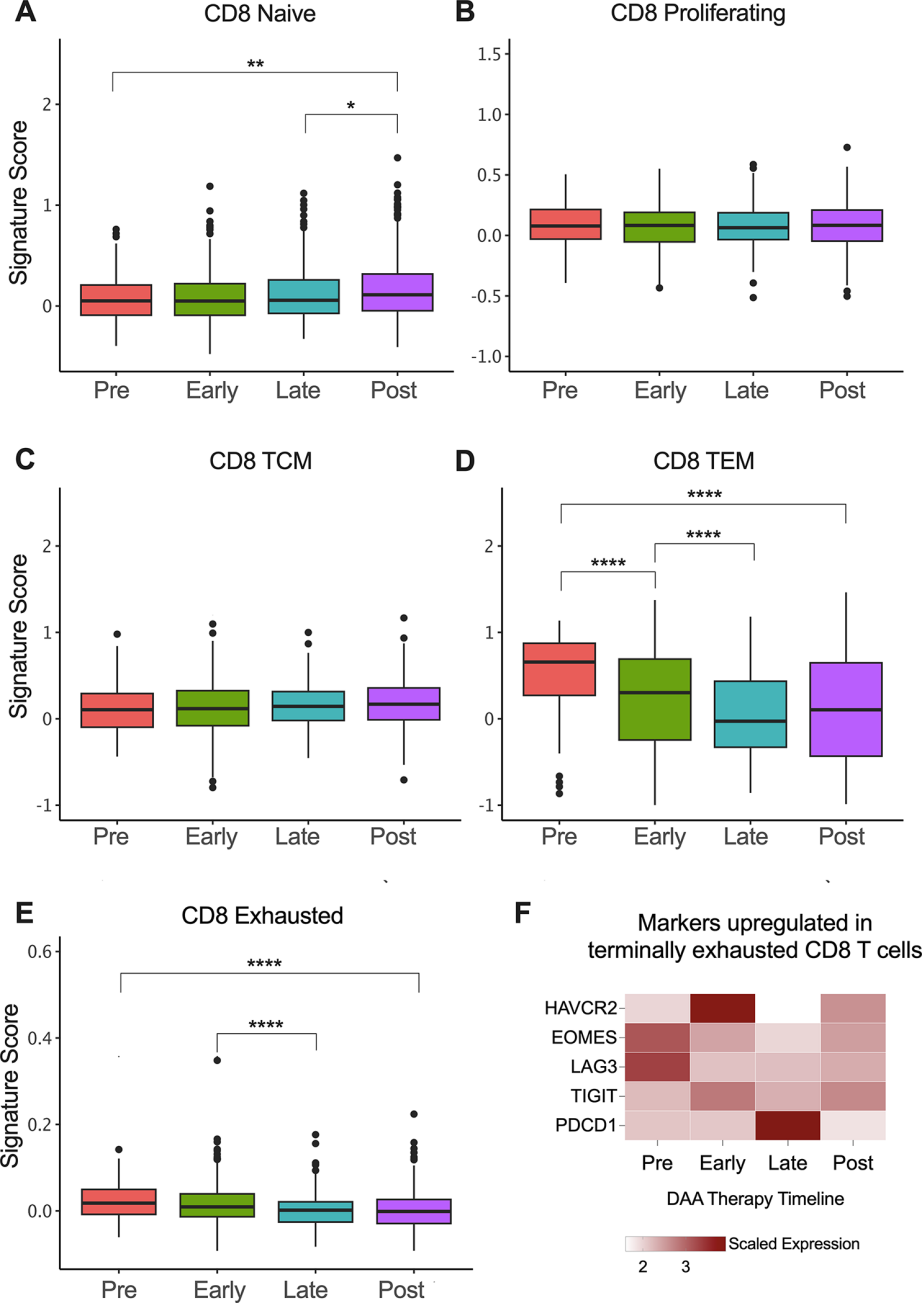
CD8^+^ T cell phenotype signatures change during DAA therapy. **(A–E)** Box plots showing the signature scores of naïve, proliferating, central memory (TCM), effector memory (TEM), and exhausted CD8+ T cells profiles. *P ≤ 0.05; **P ≤ 0.01; ***P ≤ 0.001; ****P ≤ 0.0001. **(F)** Heatmap showing the scaled expression of genes commonly upregulated in terminally exhausted CD8^+^ T cells.

We also evaluated the phenotypes of CD8^+^ T cells during DAA therapy and as seen in rHCV, there was a small but statistically significant increase in the proportion of CD8^+^ naïve T cells in successfully treated subjects (Figure 4A). We did not detect changes in A1 HCV-specific proliferating or TCM phenotypes rates during DAA therapy (Figures 4B, C). However, similar to rHCV, we detected a significant reduction in the proportion of TEM during DAA therapy (Figure 4D). We also detected a slight decrease in CD8^+^ exhausted T cells during treatment (Figure 4E). Of note, we detected a decrease in the expression of HAVCR2 (Tim-3), EOMES, and LAG3 (Figure 4F), which characterize terminally exhausted T cells.

### 3.5 Non-HCV virus-specific CD8^+^ T cells display a cytotoxic profile during chronic HCV

The impact of HCV infection on the immune defense against other viruses is not fully understood. Here we evaluated the immune status of CMV-and Flu-specific CD8^+^ T cells in cHCV and rHCV subjects in comparison to non-HCV-exposed controls (CTRs).

**FIGURE 5:**
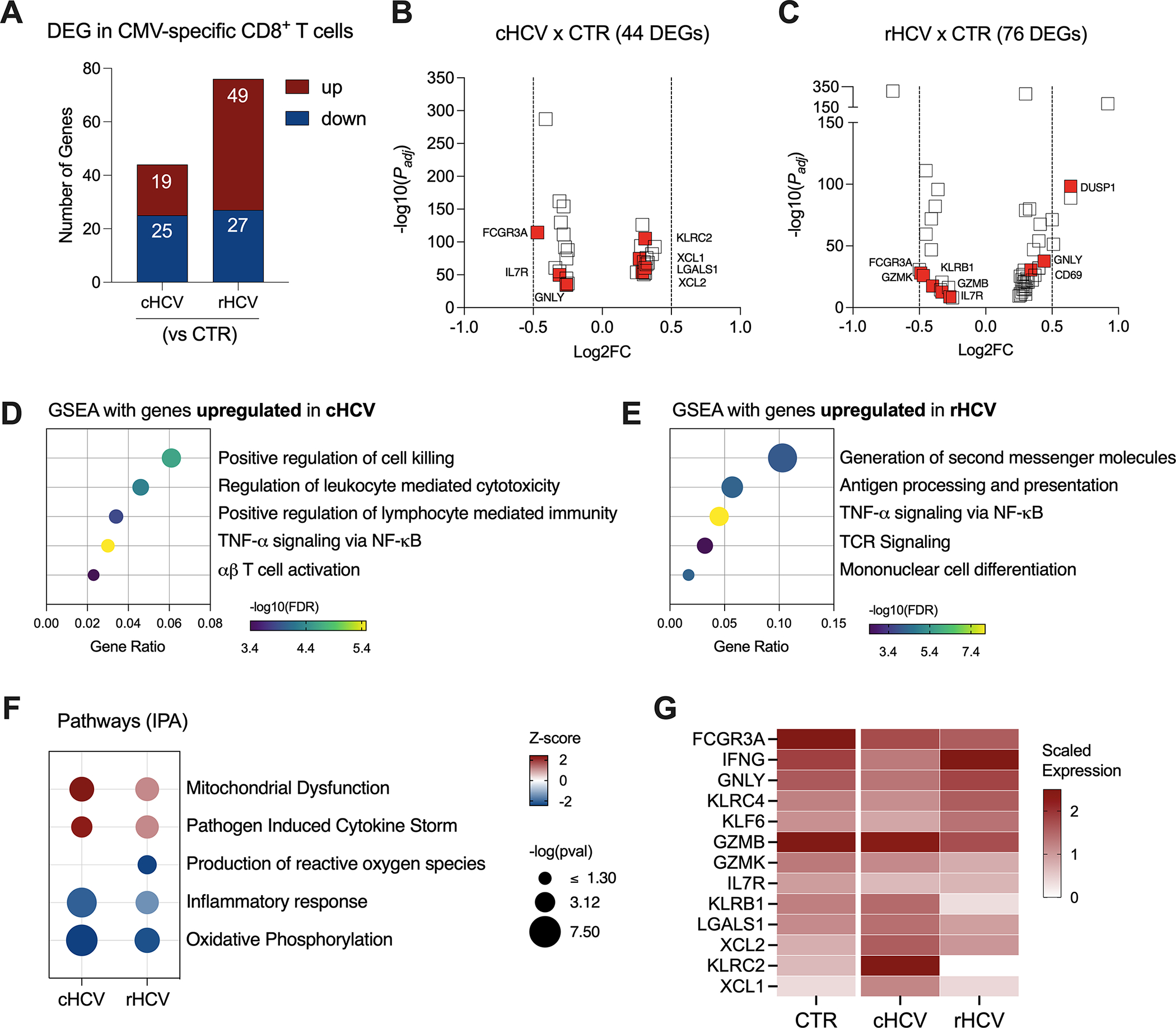
Transcriptomic analysis of CMV-specific CD8^+^ T cells isolated from chronic and resolved HCV subjects. **(A)** Differentially expressed genes identified in CMV-specific cells from cHCV and rHCV subjects in comparison to CMV-specific cells isolated from non-HCV-exposed controls. **(B, C)** Volcano plots showing the distribution of CMV-specific cells DEGs from cHCV and rHCV. **(D, E)** Plots showing the results of the Gene Set Enrichment Analysis (GSEA) analysis performed using the genes upregulated in CMV-specific cells from cHCV **(D)** and rHCV **(E)** subjects. Bubble plot size derived from gene ratio located at X-axis. **(F)** Pathways were identified using the whole DEG found in CMV-specific cells isolated from cHCV and rHCV. **(G)** Heatmap showing scaled expression of genes with immunological relevance expressed by CMV-specific cells isolated from controls, cHCV, and rHCV subjects.

We found 44 DEGs in CMV-specific T cells from cHCV subjects (19 up and 25 downregulated), and 78 DEGs (49 up and 27 downregulated genes) in rHCV subjects (Figure 5A, Supplementary Table 2). The most relevant differentially expressed genes are shown in Figures 5B, C. Additionally, we identified 105 DEGs (30 up and 75 downregulated) when comparing cHCV to rHCV directly (Supplementary Figure 3A). The 19 genes found upregulated in CMV-specific CD8^+^ T cells in cHCV (vs CTRs) enriched processes related to cell killing, positive regulation of immune response, and TNF-α signaling, suggesting a more pro-inflammatory profile (Figure 5D). The 49 genes upregulated in rHCV subjects (vs CTRs) enriched pathways controlling the generation of second messenger molecules, antigen processing and presentation, TNF-α signaling, TCR signaling, and cell differentiation (Figure 5E). We also detected an overall inhibition of oxidative phosphorylation in CMV-specific T cells in both cHCV and rHCV subjects when compared to controls (Figure 5F, Supplementary Figure 3B), with inhibition of several genes involved in the mitochondrial respiratory chain (Supplementary Figure 3C). Of note, several genes with immunological relevance were identified among the DEGs (Figure 5G, Supplementary Table 3). We found GNLY and IFNG downregulated and XCL1, XCL2, KLRC2, and LGALS1 upregulated in cHCV. GZMB, GZMK, KLRB1, and KLRC4 were downregulated in rHCV, while KLF6 was upregulated. CD127 (IL7R) and FCGR3 (CD16) were downregulated in both cHCV and rHCV compared to controls.

**FIGURE 6:**
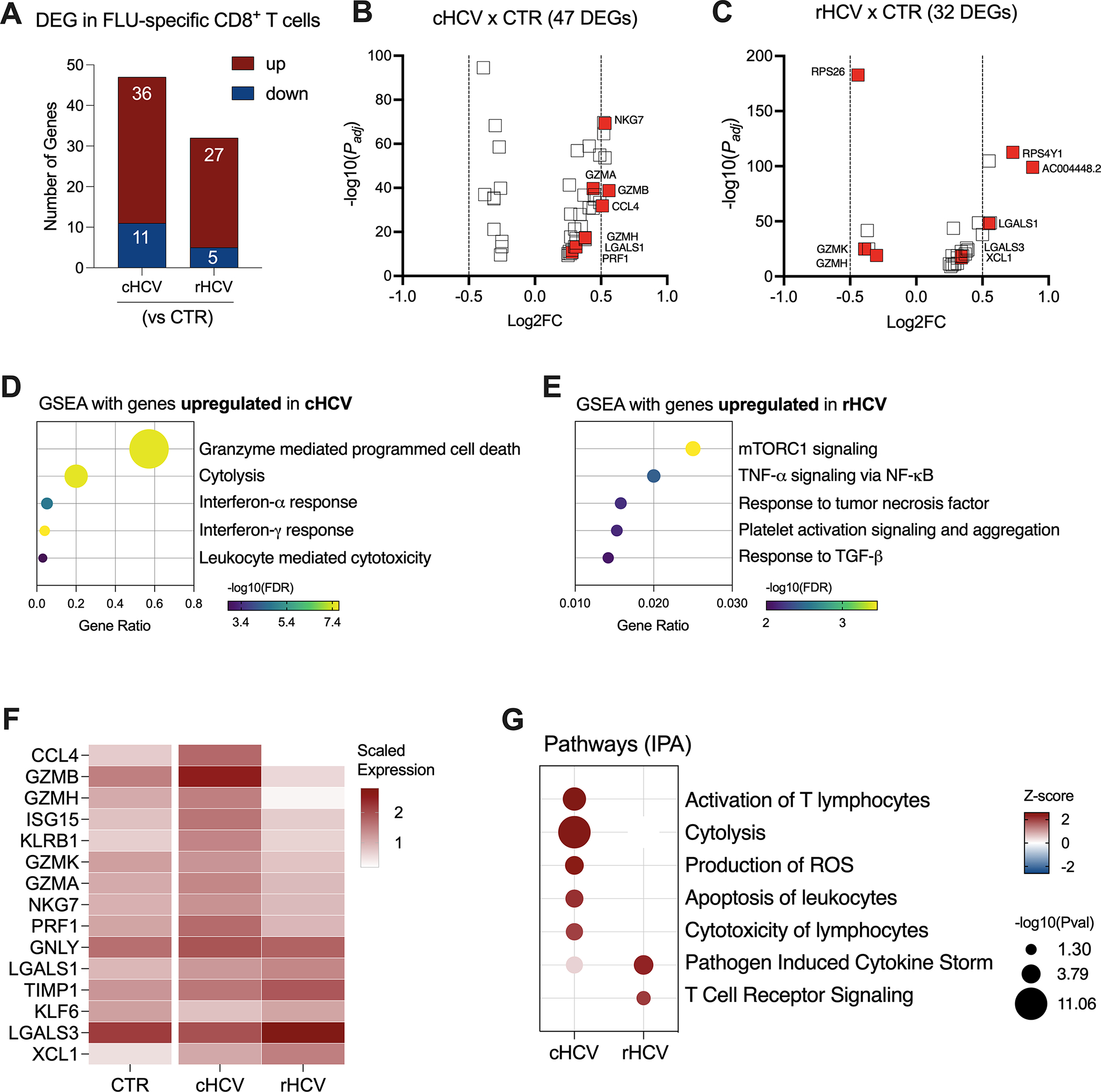
Transcriptomic analysis of Flu-specific CD8^+^ T cells isolated from chronic and resolved HCV subjects. **(A)** Differentially expressed genes identified in Flu-specific cells from cHCV and rHCV subjects in comparison to CMV-specific cells isolated from non-HCV-exposed controls. **(B, C)** Volcano plots showing the distribution of Flu-specific cells DEGs from cHCV and rHCV. **(D, E)** Plots showing the results of the Gene Set Enrichment Analysis (GSEA) analysis performed using the genes upregulated in Flu-specific cells from cHCV **(D)** and rHCV **(E)** patients. Bubble plot size derived from gene ratio located at X-axis. (F) Heatmap showing genes with immunological relevance expressed by Flu-specific cells isolated from controls, cHCV, and rHCV subjects. Gene values correspond to scaled average gene expression. **(G)** Pathways were identified using the whole DEG found in Flu-specific cells isolated from cHCV and rHCV subjects.

We found 47 DEGs in Flu-specific CD8^+^ T cells from cHCV subjects (36 up and 11 downregulated), and 32 DEGs (27 up and 5 downregulated) from rHCV cases in comparison to Flu-specific CD8^+^ T cells isolated from non-HCV-exposed controls (Figure 6A, Supplementary Table 2). In a direct comparison between cells from cHCV and rHCV, we detected 46 DEGs, where 23 genes were upregulated and 23 downregulated (Supplementary Figure 4A). The volcano plots in Figures 6B, C show the DEG distribution for cHCV and rHCV versus control. Overall, the upregulated genes of Flu-specific CD8^+^ T cells in cHCV were involved in cytotoxicity and interferon response (Figure 6D), while the genes upregulated in the rHCV subjects were associated with mTOR, TNF-α, and TGF- β signaling, and platelet activation and aggregation (Figure 6E). The downregulated genes found in Flu-specific cells from cHCV were related to oxidative phosphorylation, as observed in CMV-specific cells in cHCV (Supplementary Figure 4B). The main genes associated with this process were involved in the mitochondrial respiratory chain (Supplementary Figure 4C).

The differentially expressed genes with immunological relevance can be found in Figure 6F (Supplementary Table 3). The gene expression of CCL4, GZMA, GZMB, GZMH, GZMK, ISG15, KLRB1, NGK7, and PRF1 was upregulated in Flu-specific CD8^+^ T cells from cHCV subjects, while the genes KLF6 and LGALS3 were downregulated. In Flu-specific cells isolated from rHCV subjects, the expression of LGALS3, TIMP1, and XCL1 was upregulated, while granzymes GZMH and GZMK were downregulated.

The expression of GNLY and LGALS1 was upregulated in cHCV and rHCV. Using IPA, we found that, besides cytotoxicity, Flu-specific CD8^+^ T cells from cHCV subjects also upregulated pathways related to T cell activation, reactive oxygen species (ROS) production, and apoptosis of leukocytes, whereas cells from rHCV subjects showed upregulation of pathways involved in cytokine storm and TCR signaling (Figure 6G).

Regarding the CD8^+^ T cell phenotype signature, we observed that CMV-specific cells from cHCV subjects have low rates for naïve and TCM cells, while displaying a higher rate for TEM (Supplementary Figure 5A). No differences were observed in the proportion of CD8^+^ exhausted T cells. Flu-specific cells from cHCV subjects also demonstrated low proportions of the naïve phenotype and an increased proportion of TEM. However, in contrast to CMV-specific cells, we found a significant increase in the frequency of CD8^+^ exhausted T cells that were specific to Flu during chronic HCV infection (Supplementary Figure 5B).

## 4 Discussion

In this study, we identified significant differences between HCV-specific CD8^+^ T cells during chronic HCV infection (cHCV) and after spontaneous HCV resolution (rHCV), how the DAA therapy affects the immunological status of these cells, and how current and past HCV infections impact the function of non-HCV virus-specific CD8^+^ T cells. HCV-specific CD8^+^ T cells play a central role in the defense mechanisms against HCV infection via cytolytic activity and cytokine secretion, and their participation is pivotal for viral clearance (7, 23, 24).

In acute HCV infection (first six months), HCV-specific CD8^+^ T cells only appear in the circulation around 8–12 weeks after HCV exposure, when they are recruited to the liver by chemokines for the receptor CXCR3 and CCR5 (25). Once in the liver, HCV-specific CD8^+^ T cells will start the inflammatory phase of the disease, eventually contributing to the advancement of liver injury. In case of re-infection, HCV-specific CD8^+^ T cells enter the circulation earlier. When these cells are depleted before re-infection in the animal model, viremia is prolonged, supporting the idea that HCV-specific memory T-cells are essential for the HCV immune protection response (7, 26). While a robust and sustained response of HCV-specific CD8^+^ T cells associates with a self-limited HCV infection, a weak and transitory T cell response favors viral persistence, contributing to HCV infection chronicity (27, 28).

In chronic HCV, we found HCV-specific CD8^+^ T cells displaying cytotoxic traits with enrichment of pathways related to IFN-γ response, and IL-12 and TNF-α signaling. The subjects included in this study carried the HLA alleles A*0101 and A*0201. HLA-A*02 has been linked to HCV viral clearance and HLA-A*01 with chronic infection (29). A deeper analysis of the CD8^+^ T cells specific for different epitopes of HCV revealed that these two cell groups exhibit distinct immunological phenotypes, with the A1-specific cells displaying cytolytic features in cHCV subjects and the A2-specific cells being more involved in antigen processing and presentation, and PD-1 signaling. HCV-specific CD8^+^ T cells from spontaneously resolved HCV (rHCV) subjects showed the upregulation of genes controlling apoptosis, stress-induced senescence, and cell differentiation. Regarding the CD8^+^ T specific for different epitopes of HCV in rHCV, we found that, besides the overall features described above, the A1-specific cells also display upregulation of TNF-α and TGF-β signaling and the A2-specific cells show increased cytotoxicity. We used dextramers that would recognize the epitopes NS3 (A1_1435_) and NS5 (A2_2594_). Therefore, our findings support that the HCV proteins have a direct impact on the immune response during HCV infection.

We used the datasets published by Hao et al. and Doering et al. to annotate the virus-specific CD8^+^ T cell phenotypes (14, 15). We detected an increased rate of CD8^+^ T cells displaying a T effector memory (TEM) signature in the blood of cHCV patients and a predominance of the CD8^+^ T central memory (TCM) cells in resolved HCV subjects. TEM and TCM cells have different functions. TEM cells are preferentially found in the blood circulation, contain the spread of pathogens, and can rapidly become cytotoxic when in contact with antigen. TCM cells normally circulate between lymphoid organs and require short-term stimulation to become cytotoxic. It is possible to observe changes in the proportions of TEM and TCM populations throughout a viral infection, with a progressive increase of TCM after viral clearance and memory establishment (30). This could explain the difference in cytotoxicity observed in HCV-specific CD8^+^ T cells from cHCV and rHCV.

Several differentially expressed genes of immunological relevance were identified in the HCV-specific CD8^+^ T cells from cHCV and rHCV subjects. IL32 was significantly downregulated in A1-specific cells in cHCV. This pro-inflammatory cytokine enhances CD8^+^ T cells antitumor activity and, although it has anti-viral activity against HBV, there is no data supporting the antiviral activity of IL32 against HCV (31, 32). TGFB1 expression was overexpressed by the A1-specific cells in cHCV. T cells are an important source of TGF-β, but its signaling may lead to CD8^+^ T cell dysfunction (33). In chronic lymphocytic choriomeningitis virus (LCMV) infection, the virus-specific CD8^+^ T cells also display increased TGFB1 expression, and its signaling attenuation improves cell survival and anti-viral capacity (34). We found the chemokines CCL4 and XCL1 upregulated in cHCV. CCL4 is a chemokine that can recruit other cytotoxic T lymphocytes, and its reduction in serum levels is associated with sustained viral response in HCV patients after treatment (35, 36). XCL1 expressed by activated CD8^+^ T cells recruits XCR1^+^ dendritic cells to the infected tissue to maximize the antiviral response (37). The Kruppel-like factors (KLF) 2 and 6 were inhibited in HCV-specific cells in cHCV. The downregulation of KLF2 A1_1435_-specific CD8^+^T cells from cHCV subjects results in the observed upregulation of CXCR3. It is known that persistent TCR triggering results in the loss of KLF2 expression, which favors CD8^+^ T cells fully achieving effector differentiation, with consequent upregulation of CXCR3 expression in this cell phenotype (38). The expression of CXCR3 in activated effector CD8^+^ T cells is essential for CD8^+^ T cell migration to inflammatory sites (39). Other authors also reported an increase of intrahepatic T lymphocytes overexpressing CCR5 and CXCR3 during chronic HCV (40, 41). KLF2 re-expression, as observed in resolved HCV, would favor the conversion of effector CD8^+^ T cells into memory cells. KLF6 is a tumor suppressor gene associated with several types of cancer. In T cells, KLF6 is involved in the differentiation, activation, and homing of various T-cell subsets, and regulates iNOS production under stress (42). Our dataset also showed that CD127 (IL7R) was downregulated in A1 and A2 HCV-specific CD8^+^ T cells from chronic HCV patients in comparison to rHCV subjects. This finding corroborates previous observations that CD127 expression is lower on CD8^+^ T cells specific for persistent viruses, including HCV (43, 44).

Previously, we had shown that DAA therapy induces a rapid normalization of IFN signaling in circulating CD3^+^ T cells (12). In this study, we assessed the impact of DAA therapy on HCV A1_1435_-specific CD8^+^ T cells. Here, we showed that the first effect of the DAA therapy on these cells was a significant cytotoxicity reduction, which lasted until 12 weeks after the DAA regimen completion. We also observed a fluctuation in the expression of IFNG, FASLG, and TNF during the DAA therapy. Before therapy, cells showed low expression of IFNG and FASLG. However, in the early phases of the therapy, we detected a peak of IFNG expression, that was back to the baseline levels as the therapy continued. TNF expression was downregulated in later phases of the therapy. IFN-γ and TNFα secretion by CD8^+^ T cells is involved in noncytolytic HCV clearance by inhibiting the viral replication without killing the infected cell (45). On the other hand, FASLG is part of the cytolytic FAS-FASL system used by CD8^+^ T cells to kill HCV-infected hepatocytes (46). During the treatment, we also noticed a small but significant increase in cells displaying CD8^+^ naïve T cell signature, as well as a decrease in cells expressing CD8 T effector memory and exhaustion markers, which may be correlated to the HCV clearance promoted by DAA. In addition, we also compared the pathways important for A1_1435_-specific CD8^+^ T cells from resolved HCV subjects and from DAA-treated patients. Both groups showed inhibition of pathways related to cytotoxicity. However, cells from rHCV cases showed upregulation of TCR signaling, while HCV-specific CD8^+^ T cells from DAA post-therapy showed a remarkable increase in RNA translation. The cell translation machinery directly responds to changes in the environment so the cell can conserve energy and reprogram gene expression and signaling pathways to restore homeostasis. In LCMV infection, memory precursor LCMV-specific CD8^+^ T cells showed an increase of differentiation to terminal effector CD8^+^ T cells, suggesting that this process may be important for the survival and generation of T memory cells (47).

We also investigated the immunological status of non-HCV virus-specific CD8^+^ T cells on the background of HCV infection. Although CMV can infect practically all organ tissues, it can cause hepatitis in immunocompromised patients (48). Co-infection of CMV and HCV has been reported, with rates of 36% among cHCV patients and 14% in rHCV subjects (49, 50). Additionally, chronic HCV patients co-infected with CMV showed poor response to interferon and ribavirin therapy and worsening liver fibrosis (51). CD8^+^ T cells specific for CMV can recognize several virus epitopes. However, the cells specific for epitope pp65, commonly detected in HLA A2-positive donors, show significantly higher functionality (52). Here, we profiled CMV(A2-pp65)-specific CD8^+^ T cells in chronic and resolved HCV infection and compared them to CMV-specific cells isolated from non-HCV-exposed subjects. Our findings revealed that CMV-specific cells display a cytotoxic profile, with overexpression of granzyme B, and pro-inflammatory features, such as overexpression of the dendritic cell chemoattractants XCL1 and XCL2 in cHCV patients, suggesting that CMV-specific CD8^+^ T cells may become activated during a current HCV infection and contribute to liver disease progression, especially in cases of HCV and CMV co-infection. Influenza (Flu) is a respiratory virus that does not have tropism for hepatic cells. However, hepatocellular injury can be caused by moderate hypoxia resulting from respiratory illness (53). Flu-specific CD8^+^ T cells from cHCV patients, similarly to CMV, displayed a cytotoxic and pro-inflammatory profile, which indicates that these cells may also contribute to the progression of hepatitis caused by HCV. Of note, we detected an increased CD8^+^ exhausted T cell signature in Flu-specific cells in cHCV subjects, which suggests that these cells become dysfunctional during chronic HCV infection. Similar heterologous cross-reactivity has already been demonstrated in human and murine studies (54). Flu-specific memory T cells from HCV-negative blood donors can expand and become cytotoxic *in vitro* when stimulated by HCV_NS3–1073_ antigen, and HCV-specific T cells could be induced *in vivo* by Flu infection. Therefore, our findings suggest that the cytotoxic and pro-inflammatory profile observed in CD8^+^ T cells specific to other non-HCV viruses may be a bystander effect of the current chronic HCV infection and support the idea that HCV infection alters the overall adaptive immune response of the affected patients.

Although our study sheds new light on the effects of chronic HCV infection on CD8^+^ T cells regardless of virus specificity, it does however have some limitations. Due to the small number of virus-specific CD8^+^ T cells obtained from each subject (ranging from 300 to 1,200 cells), we cannot perform functional assays to validate the cell cytotoxicity suggested by the single-cell transcriptomic analysis. Moreover, the experimental costs (scRNAseq + barcoded dextramers) limited our cohort size and the number of epitopes we could include in this study. Thus, we selected epitopes with immunogenic response in our cohort that have been previously used as antigens for other T-cell response studies. Therefore, the use of different CMV and Flu epitopes presented by different HLA alleles could also result in contrasting CD8^+^ T cell responses, as we observed in HCV-specific cells. Nevertheless, our data provide useful information to groups exploring the underlying mechanisms of HCV-specific T-cell spontaneous and treatment-induced resolution.

Collectively, our data demonstrate that virus-specific CD8^+^ T cells exhibit robust cytotoxic traits during chronic HCV infection, regardless of the targeted virus when compared to the same cell population from rHCV cases, that CD8^+^ T cells specific for different HCV epitopes demonstrate distinct immunological profiles and that DAA therapy inhibits HCV-specific CD8^+^ T cell cytotoxicity.

## Supplementary Material

Supplementary Material

Supplementary table 1

Supplementary table 3

Supplementary table 2

## Figures and Tables

**TABLE 1:** Dextramer^®^ information (Immudex).

Dextramer Information					
#	Allele	Peptide	Category	Cat#	Epitope
1	HLA-A*0101	ATDALMTGY	HCV	WA03541	NS3:1435–1443
2	HLA-A*0201	ALYDVVTKL	HCV	WB03542	NS5:2594–2602
3	HLA-A*0201	NLVPMVATV	CMV	WB02132	pp65:495–504
4	HLA-A*0201	GILGFVFTL	Influenza	WB02161	MP 58–66
5	HLA-B*0801	*random*	Neg. Control	WI03233	*random*
6	HLA-A*0101	*random*	Neg. Control	WA03579	*random*

**TABLE 2:** CD8^+^ T cell phenotype signature genes.

Label	Markers	Reference
CD8^+^ Naive T	CD8B, S100B, CCR7, RGS10, NOSIP, LINC02446, LEF1, CRTAM, CD8A, OXNAD1	Hao et al. (14)
CD8^+^ Proliferating T	MKI67, CD8B, TYMS, TRAC, PCLAF, CD3D, CLSPN, CD3G, TK1, RRM2	Hao et al. (14)
CD8^+^ Central Memory T	CD8B, ANXA1, CD8A, KRT1, LINC02446, YBX3, IL7R, TRAC, NELL2, LDHB	Hao et al. (14)
CD8^+^ Effector Memory T	CCL5, GZMH, CD8A, TRAC, KLRD1, NKG7, GZMK, CST7, CD8B, TRGC2	Hao et al. (14)
CD8^+^ Exhausted T	ABCC2, ABTB2, ADAMTS2, ADAR, ADM, ADORA2A, AIM2, AMPD3, ANKLE2, APOBEC3A, APOL1, ARAP2, ARHGAP27, ASAP2, B4GALT5, BAALC, BAZ1A, BHLHE22, BIRC2, BLVRA, BLZF1, LINC01588, C15orf32, SHFL, C3orf38, C4orf46, CPLANE1, SNHG15, CCL4, CCL5, NOCT, CD40, CD83, CDC14C, CDKN2B, CELA1, CFLAR, CLEC2D, CPNE8, CSRNP1, CXCL9, CXorf65, DAPP1, DDX58, DDX60L, DGKH, DMRT2, DNAAF1, DUSP5, EGFL7, EGFLAM, EHD1, EHD4, EPSTI1, EREG, ERRFI1, ESPL1, FAM174B, FBP2, FBXO40, FCGR1BP, ENSG00000280119, FUT4, FXYD6, GCH1, GJB2, GNGT1, GPR183, GPR32, GRHL1, GTPBP1, HERC5, HERC6, HSF4, IDO2, IFI35, IFIH1, IL18R1, IL18RAP, IL19, IL6, IL7R, IMPG1, ISX, KCNJ2, WDR97, KRT17, KRT80, LAG3, SIPA1L1, LINC01785, ENSG00000216775, MIR3945HG, LYN, MAP2K3, MAP3K8, MAP4K4, MASP1, MCTP1, MEST, MIR155HG, MX2, MXD1, MYO1A, NAMPT, NCF1C, NEDD4L, NINJ1, NPY1R, NXF1, OAS1, OAS3, OASL, OTUD4, P2RX4, P2RX7, PALM3, TENT4A, PARP14, PELI1, PI4K2B, PIK3R3, PIK3R5, PIM3, PIWIL4, PLEK, PLK3, PML, POPDC2, PPFIBP2, PPP1R15A, PRG3, HELZ2, PRSS23, PRX, PSD3, PTGER2, PTGS2, PTK2B, PTPRF, PVR, NECTIN3, RAB24, RBBP6, RBMXL1, REN, RIPK1, RIPK2, RNF144B, RNF19A, RNF213, SAMD9L, SCGB2A1, SDC4, SERPINB1, SERPINE1, SKIL, SLAMF1, SLAMF7, SLC1A2, SLC1A3, SNN, SORBS1, SPAG1, SPATA6, STARD5, STAT1, STAT5A, STX11, TAAR5, TDRD7, TNS2, TNIP2, TNIP3, TP53INP2, TRIL, TRIM22, TSHB, TSIX, TSPO2, TTN, TYSND1, UBE2L6, USP18, UST, VCAN, DNAI3, WTAP, XRN1, ZBTB10, ZC3HAV1, ZNFX1	Doering et al. (15)

## Data Availability

The datasets presented in this study can be found in online repositories. The name of the repository and accession number can be found below: NCBI; GSE228763.
