# Supplementary material for: Chronic HCV infection promotes cytotoxicity in antigen-specific CD8+ T cells regardless of virus specificity
Source: Front Virol. Author manuscript; Available in PMC 2023 Oct 26. (PMC10601542; doi:10.3389/fviro.2023.1198361)
Supplement: Supplementary Material [file NIHMS1932725-supplement-Supplementary_Material.docx]

Supplementary Material

Chronic HCV infection promotes cytotoxicity in antigen-specific CD8^+^ T cells regardless of virus specificity.

Ana C. Maretti-Mira^1,2*^, Matthew P. Salomon^1^, Angela M. Hsu^1,2^, Chikako Matsuba^1^, Lucy Golden-Mason^1,2^

^1^USC Research Center for Liver Diseases, Keck School of Medicine, University of Southern California, Los Angeles, CA.

^2^Division of Gastrointestinal and Liver Diseases, Department of Medicine, Keck School of Medicine, University of Southern California, Los Angeles, CA.

***Correspondence:**

Ana C. Maretti-Mira

E-mail: [marettim@usc.edu](mailto:marettim@usc.edu)

# Content List:

Supplementary Figure 1

Supplementary Figure 2

Supplementary Figure 3

Supplementary Figure 4

Supplementary Figure 5


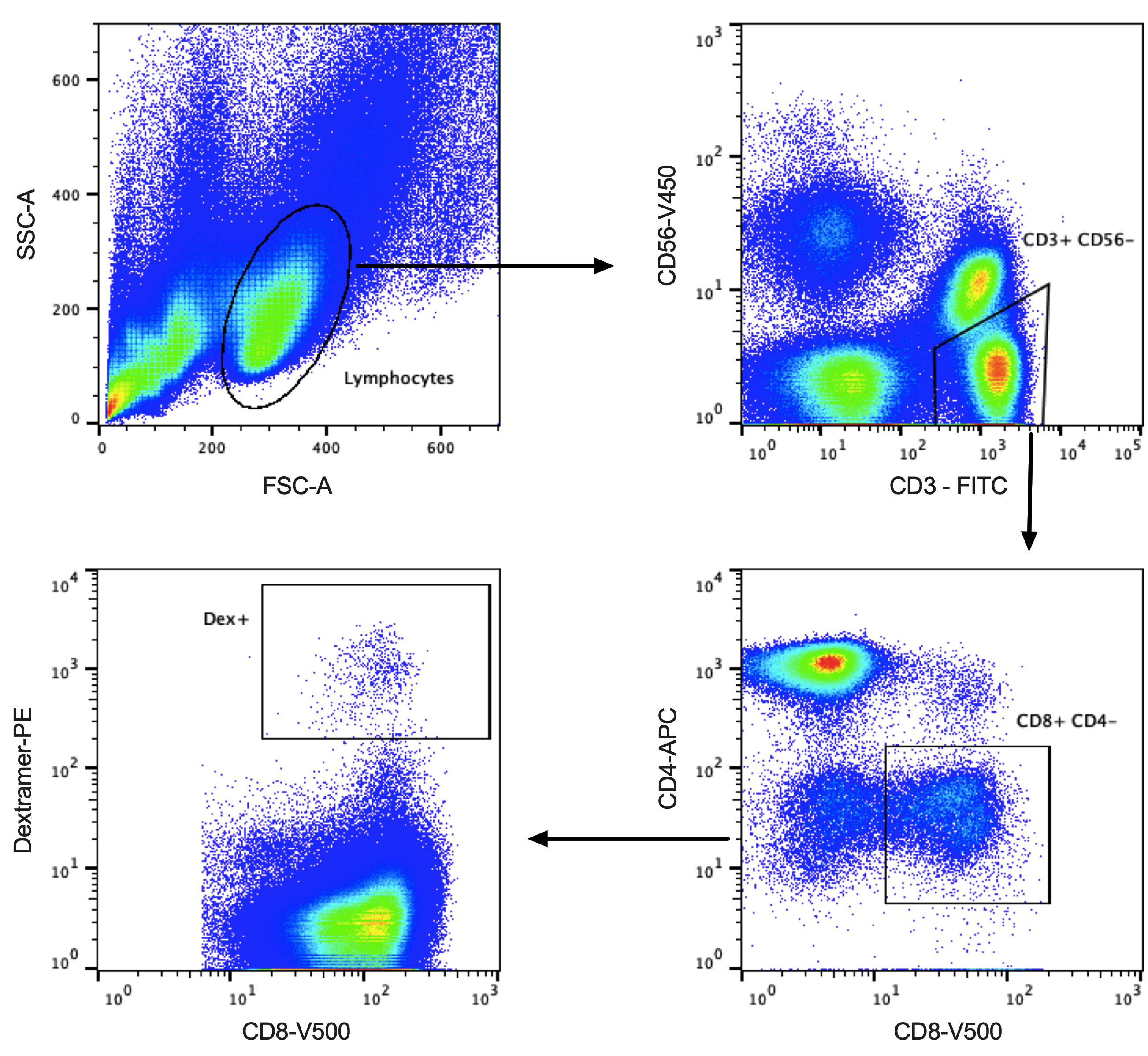


**Supplementary Figure 1.** Sorting strategy for isolation of virus-specific CD8^+^ T cells. Representative graphics from FACS sorting showing the gating strategy used to sort Dex^+^ CD8^+^ (CD3^+^ CD56^-^ CD4^-^) T cells from peripheral blood mononuclear single cell suspensions. Cells were sorted in 90% FBS solution at 4˚C. No Dex^+^ cells were detected in CD56^+^CD8^+^ T cells.


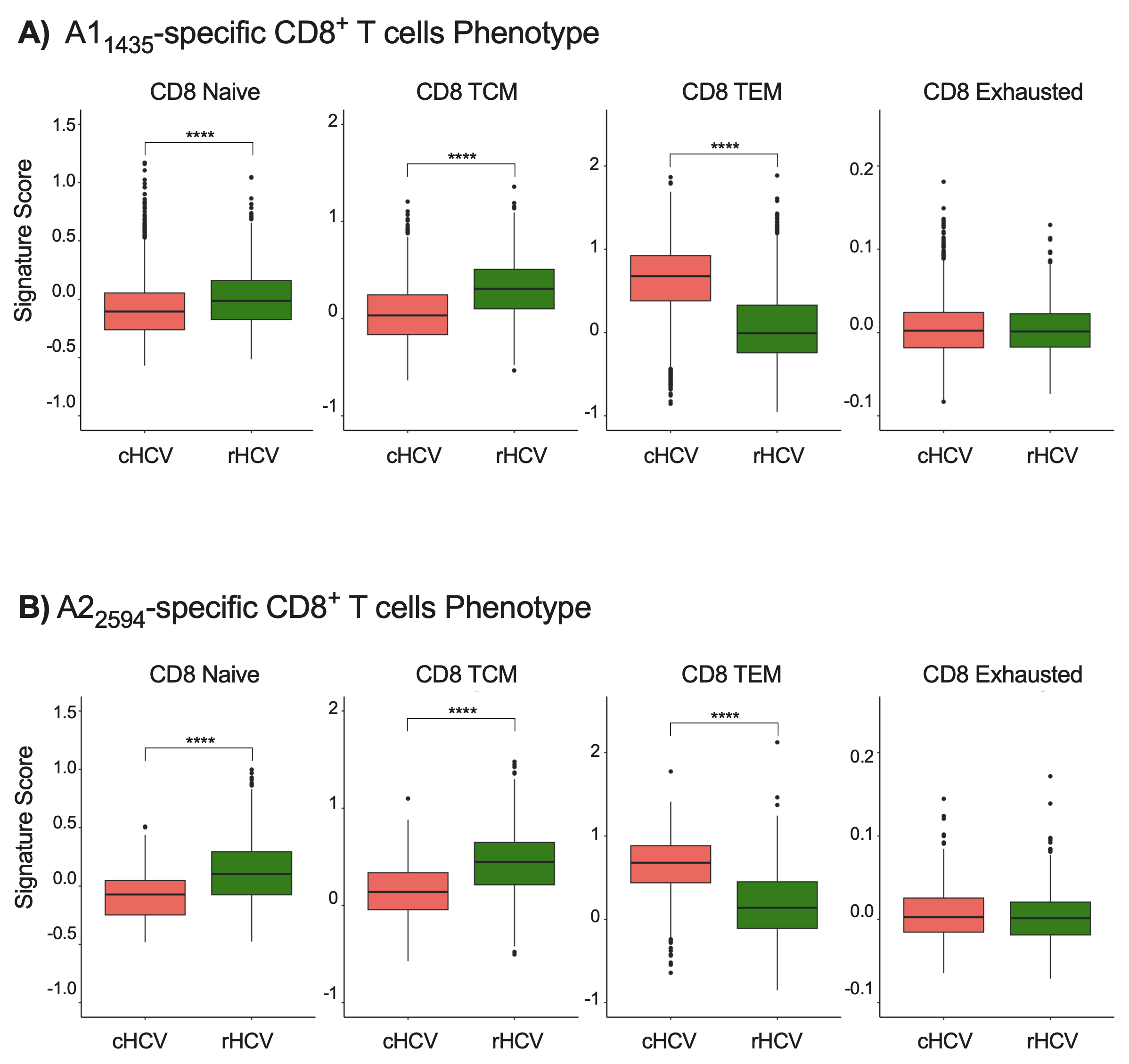


**Supplementary Figure 2.** CD8^+^ T cell phenotype signatures of HCV-specific cells. Box plots showing the signature scores of naïve, central memory (TCM), effector memory (TEM), and exhausted CD8^+^ T cell profiles in A11435-specific CD8^+^ T cells **(A)** and A22594-specific CD8^+^ T cells **(B)**. (Mann-Whitney U, ****P ≤ 0.0001)


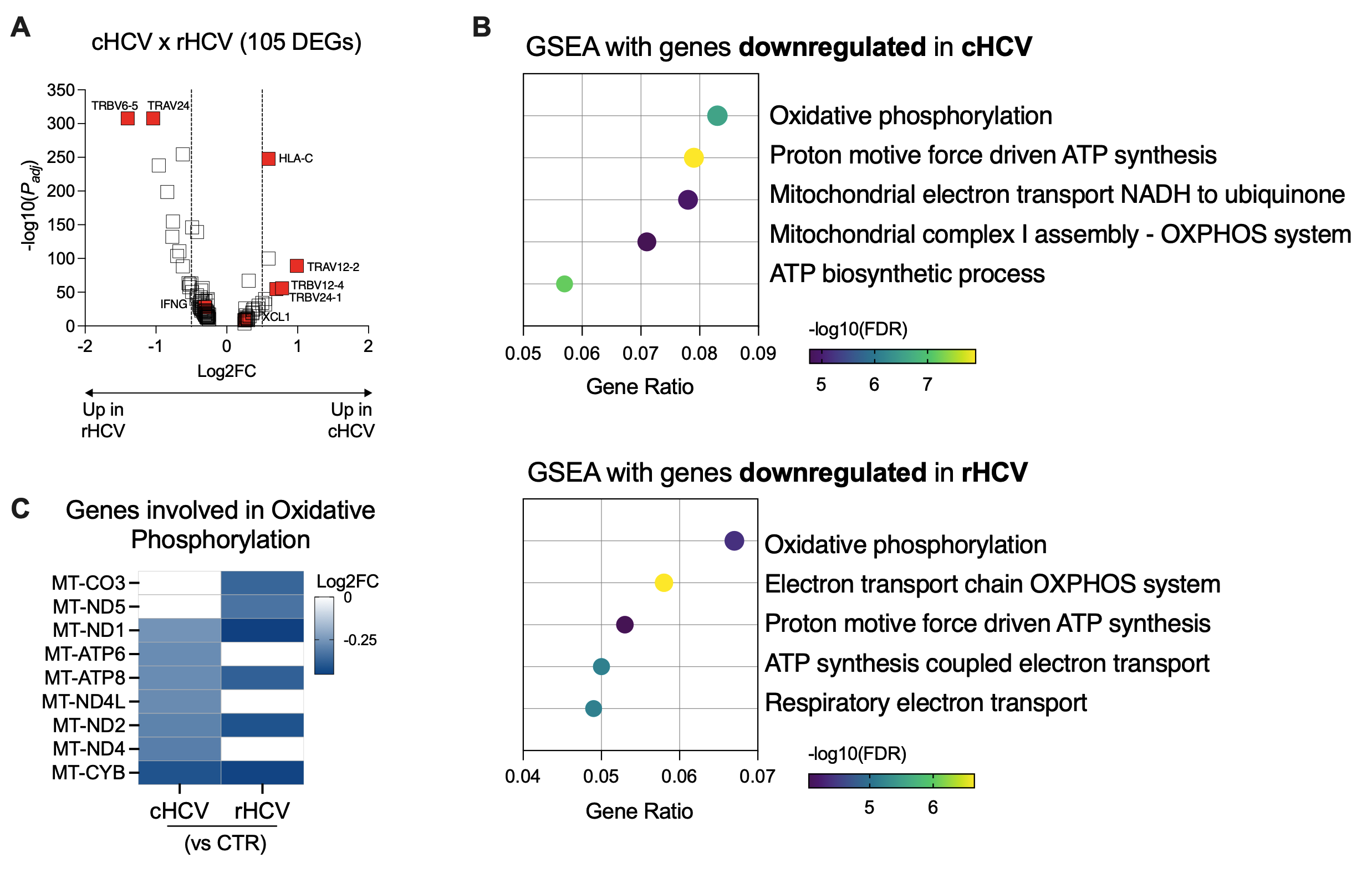


**Supplementary Figure 3.** Transcriptional changes observed in CMV-specific CD8^+^ T cells isolated from cHCV and rHCV. **(A)** Volcano plot showing the DEG obtained from the comparison between cHCV and rHCV. **(B)** Plots displaying the most relevant GSEA terms from the enrichment analysis performed with genes downregulated in CMV-specific cells isolated from cHCV and rHCV. **(C)** Heatmap showing the fold changes of genes involved in oxidative phosphorylation expressed in CMV-specific CD8^+^ T cells.


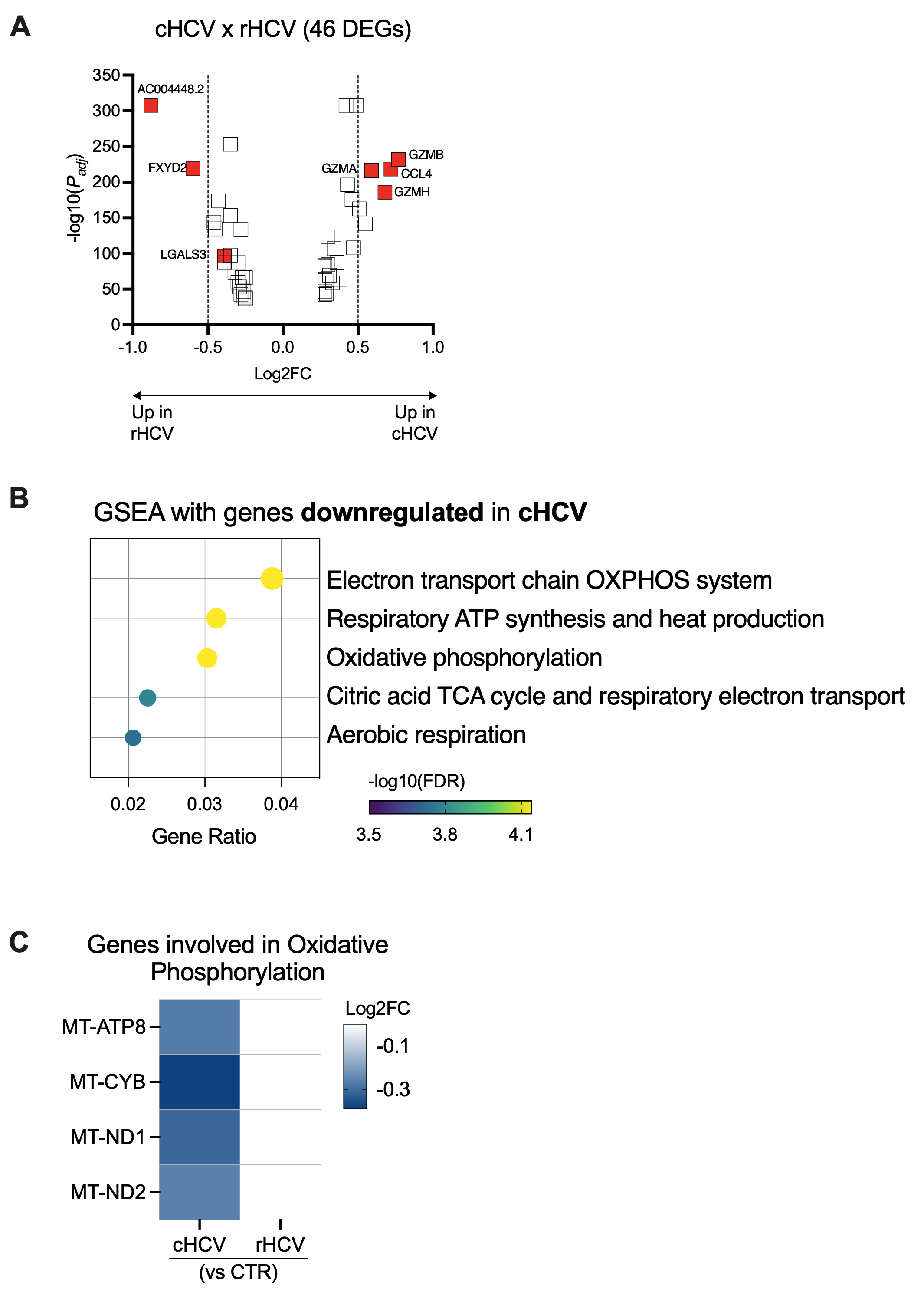


**Supplementary Figure 4.** Transcriptional changes observed in Flu-specific CD8^+^ T cells isolated from cHCV and rHCV. **(A)** Volcano plot showing the DEG obtained from the comparison between cHCV and rHCV. **(B)** Plots displaying the most relevant GSEA terms from the enrichment analysis performed with genes downregulated in Flu-specific cells isolated from cHCV. Of note, we could not detect significant enrichment using genes downregulated in rHCV. **(C)** Heatmap showing the fold changes of genes involved in oxidative phosphorylation expressed in Flu-specific CD8^+^ T cells.


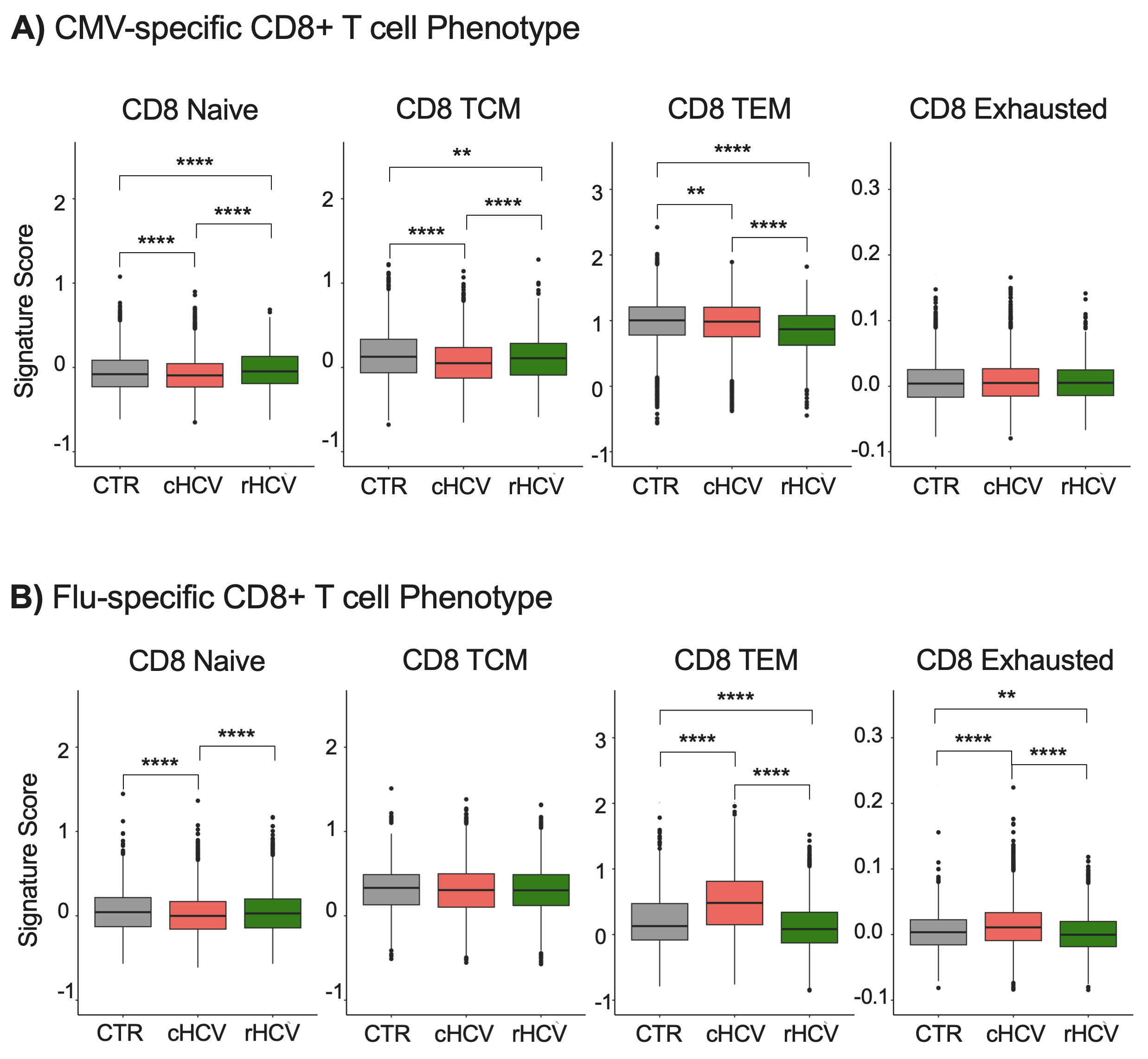


**Supplementary Figure 5.** CD8^+^ T cell phenotype signatures of virus-specific cells in cHCV and rHCV subjects. Box plots showing the signature scores of naïve, central memory (TCM), effector memory (TEM), and exhausted CD8^+^ T cell profiles in CMV-specific CD8^+^ T cells **(A)** and Flu-specific CD8^+^ T cells **(B)**. (Mann-Whitney U, **P ≤ 0.01; ****P ≤ 0.0001).
